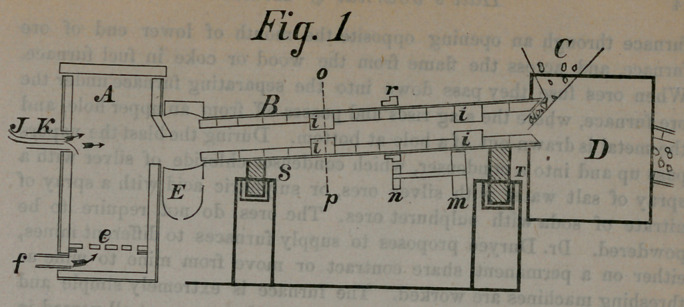# Duryees’ Blow-Pipe Ore Furnace

**Published:** 1879-08

**Authors:** 


### DURYEE'S BLOW PIPE ORE FURNACE.

### DESCRIPTION.

*A*—Fuel Furnace to generate initial fire.
*B*—Revolving Ore Furnace.
*C*—Hopper for Feeding Ores.
*D*—Condenser for Condensing Vapors.
*E*—Separating Furnace or Pot.
*f*—Blast for Fuel Furnace.
*K*—Blow Pipe Blast.
*J*—Pipe for Feeding Petroleum.
*S* and *T*—Rollers for Ore Furnace to revolve on.
*M* and *N*—Cogs to revolve Ore Furnace.
*r*—Cogs on Ore Furnace.

A new discovery, or rather the application of an old one, was seen by us at Rahway, New Jersey, last week, where Dr. G. Duryee had working an ore furnace on the principle of the blow-pipe; and when we tell our readers we saw horse-shoes melted in ten minutes from the time they were thrown into the furnace, and hard gold Pyrites quartz rock in about the same time completely desulphurizing the same, an idea of its intense heat may be formed. The quartz carried a large proportion of sulphuret of iron with the gold, called rebellious ores, because of the impossibility of working them by the present methods. With this heat we see no reason why the thousands of tons of tailings in Virginia or Western mines cannot be profitably worked. We formerly had considerable experience in sulphuret ores, and think that this furnace has solved the problem.

The sulphur in the ores with this furnace is made to supply a large proportion of the required fuel. Imagine an inclined revolving smoke-stack in nearly horizontal position, the ores shoveled in a hopper, which conducts them into the upper end, an intense blow-pipe flame entering in at lower end to meet the descending ores, and you see the working of the furnace. Flame is generated from an upright fuel furnace, which is brought up to a red heat, when an air blast two to four inches in diameter, fed with a jet of petroleum, passing in with air, the blast converting it into an inflammable gas of intense power, driven into the ore furnace through an opening opposite the mouth of lower end of ore furnace, and across the flame from the wood or coke in fuel furnace. When ores fuse they pass down into the separating furnace under the ore furnace, where the slag rises and passes off from an upper hole, and the metal is drawn out of a hole at bottom. During the blast the vapors pass up and into a condenser, which condenses chloride of silver with a spray of salt water with silver ores, or sulphuric acid with a spray of nitrate of soda with sulphuret ores. The ores do not require to be powdered. Dr. Duryee proposes to supply furnaces to different mines, either on a permanent share contract or move from mine to mine as threshing machines are worked. The furnace is extremely simple and portable—can be set up in a few hours—and any one at all versed in smelting can work it. It is well known that if ores are fused and made limpid, as we saw them, their separation from base metal or slag is an easy matter.

The capabilities of this smelter are immense, and we predict will work a revolution in the business. For glass furnaces or pottery burning this principle may be applied successfully. The blast we saw entering the ore furnace would generate steam fast enough to run a steamship of 2,000 tons. The petroleum was used at the rate of two gallons an hour. It looks as though the cheap and rapid evaporation of water and conversion into steam was brought to perfection. Dr. D. is a thorough student and chemist, and predicts that a blow pipe blast on his principle passing the length of fifty feet, through a six-inch gas pipe or flue surrounded by water in twelve inch boiler or pipe, could be kept at a pressure of five hundred pounds ; working more economically as to space for fuel and boiler room than is possible by any other means. What if fifty barrels petroleum and fifty tons of coal should do what five hundred tons coal now do, for instance, in a Cunard steamer ? There is already quite a stir in scientific and mining circles in regard to this new furnace.

This is not the sulphur burning furnace the Londoners are talking about, but a new application of an old theory ; and the only wonder is that it has never been applied before, when it is well known that the blow pipe heat is four times as intense as the same flame burning naturally.

We can vouch for the integrity and responsibility of Dr. Duryee. We have been acquainted with him for many years. Our readers who wish further information can address him at his New York office, 176 Broadway. He has a ten ton furnace practically working here on ores, to demonstrate its capacity. With Manganesse ores, the furnace can convert iron ores into steel at a single operation ; this and many other claims are being patented. Iron ores shoveled into upper end of a ten-foot cylinder with fluxes and coming out pig iron in a continuous stream, can be shown any time ores are furnished.

## Figures and Tables

**Fig. 1 f1:**